# Somatostatin and opioid receptors do not regulate proliferation or apoptosis of the human multiple myeloma U266 cells

**DOI:** 10.1186/1756-9966-28-77

**Published:** 2009-06-07

**Authors:** Céline Kerros, Thibault Cavey, Brigitte Sola, Philippe Jauzac, Stéphane Allouche

**Affiliations:** 1 Laboratoire de biologie moléculaire et cellulaire de la signalisation, UPRES-EA 3919, IFR 146 ICORE, Université de Caen, 14032 Caen, France

## Abstract

**Background:**

opioid and somatostatin receptors (SSTRs) that can assemble as heterodimer were individually reported to modulate malignant cell proliferation and to favour apoptosis. Materials and methods: SSTRs and opioid receptors expression were examined by RT-PCR, western-blot and binding assays, cell proliferation was studied by XTT assay and propidium iodide (PI) staining and apoptosis by annexin V-PI labelling.

**Results:**

almost all human malignant haematological cell lines studied here expressed the five SSTRs. Further experiments were conducted on the human U266 multiple myeloma cells, which express also μ-opioid receptors (MOP-R). XTT assays and cell cycle studies provide no evidence for a significant effect upon opioid or somatostatin receptors stimulation. Furthermore, neither direct effect nor potentiation of the Fas-receptor pathway was detected on apoptosis after these treatments.

**Conclusion:**

these data suggest that SSTRs or opioid receptors expression is not a guaranty for an anti-tumoral action in U266 cell line.

## Background

Multiple myeloma (MM) is a malignant hemopathy caused by the accumulation of slow proliferating and apoptosis-resistant cells in the bone marrow [1]. This pathology represents 10% of haematological malignancies [2] and accounts for 2% of cancer deaths per year in occidental countries [3]. Interactions between MM and the bone-marrow environment play a major role in the development of the disease and resistance to therapies [4]. Such interactions involve integrins and adhesion molecules which promote cytokines and growth factors release. After binding to their respective receptors, these factors activate diverse signal transduction pathways: MAPK (Mitogen-Activated Protein Kinase), JAK (Janus kinase)/STAT3 (signal transducers and activators of transcription) and PI3K (Phosphoinositide 3-kinase)/Akt), leading to apoptosis resistance, survival and proliferation [4]. Thus, pharmacological modulation of such pathways would represent complementary therapeutic strategies to conventional treatment for MM, which still remains incurable.

Somatostatin (Sst) is a small neuropeptide acting through a family of five G protein-coupled receptor (GPCR) subtypes 1–5 (SSTR1-5), which are expressed in lymphoid cells, the nervous and gastro-entero-pancreatic systems [5-7]. Autoradiography analysis using iodinated Sst analogs revealed that central and peripheral lymphoid organs express SSTRs [8], data that were further confirmed by RT-PCR (see for review [9]). Beside its physiological functions, Sst was revealed as a potent anti-tumoral agent, especially in neuroendocrine tumours [10,11]. For instance, protease-resistant Sst analogs such as octreotide have been successfully used for tumours treatment [11,12]. Other GPCRs than SSTRs [13-15] such as opioid receptors were demonstrated to be expressed in the immune system, to have an anti-tumoral activity [16] and to heterodimerize with SSTRs [16,17]. So, in the present study, we evaluated the potential role of somatostatin and opioid receptors in the regulation of cell proliferation and apoptosis in malignant hemopathies.

## Methods

### Cell culture

Except for the SK-N-BE and MCF-7 cells, that were cultured in Dulbecco's modified Eagle's medium (DMEM) (Sigma-Aldrich, St Louis, MO), supplemented with 10% (v/v) foetal calf serum (FCS) (BioWest), 1% (v/v) antibiotic-antimycotic mixture (Sigma, St Louis, MO), and 2 mM L-glutamine, the other cell lines were grown in RPMI 1640 + GlutaMAX (Invitrogen) supplemented with 10% (v/v) FCS and 1% (v/v) antibiotic-antimycotic mixture, all maintained at 37°C in 5% CO_2_. Twice a week, cells were counted, the viability was determined using trypan blue staining and the culture medium was replaced.

### RT-PCR

Total RNAs were extracted using the RNAgents^® ^Total RNA Isolation System (Promega) according to Chomczynski and Sacchi [18]. cDNAs were synthesized from 2 μg of RNA in a buffer supplied with the reverse transcriptase (RT) (Promega) containing 900 μM dNTP (Amersham), 20 units RNAsine (Promega), 500 ng random primers (Promega) and 200 units of Moloney murine leukaemia virus RT in a final volume of 20 μL. PCRs were performed using 2 μL of cDNAs in the PCR buffer supplied with the Taq polymerase supplemented with 1.5 mM MgCl_2_, 0.2 mM of dNTP, 2.5 units of Taq polymerase (Bioline), and 0.5 μM of each sense and antisense primer. Primers for SSTR1, 2 and 3 were chosen from a previous study [19], primers for SSTR4 and 5 and opioid receptors (KOP-, DOP- and MOP-R) were designed using primer 3 input [20]. Their sequences are listed in Table 1. PCR products were run on a 1.5% agarose or 2% NuSieve^® ^agarose gel with a 100 bp marker (Invitrogen) and stained with ethidium bromide.

**Table 1:** Primers used for SSTRs, opioid receptors and β-actin amplification by PCR

**Gene name**	**Primers**	**Cycles**	**Denaturation step**	**Elongation step**	**Anneling step**
β-actin	F – 5'ATGGATGATGATATCGCCGCG3' R-5'TCCAGACGCAGGATGGCATGG3'	35	1 min at 95°C	1 min at 72°C	1 min at 60°C

SSTR1	F-5'AGCCGGTTGACTATTACGCC3' R-5'GCTCTCACTTCTACCATTGTC3'	45	1 min at 95°C	2 min at 72°C	1 min at 60°C

SSTR2	F-5'GGTGAAGTCCTCTGGAATCC3' R-5'CCATTGCCAGTAGACAGAGC3'	45	30 sec at 95°C	2 min at 72°C	1 min at 63°C

SSTR3	F-5'TCATCTGCCTCTGCTACCTG3' R-5'GAGCCCAAAGAAGGCAGGCT3'	45	30 sec at 95°C	2 min at 72°C	1 min at 65°C

SSTR4	F-5'CACCAGCGTCTTCTTCTCA3' R-5'ATGGGGAGAGTGACCAACAG3'	35	1 min at 95°C	1 min at 72°C	1 min at 55°C

SSTR5	F-5'TCATCTGCCTGTGCTACCTG3' R-5'GGAGAGGATGACCACGAAGA3'	35	1 min at 95°C	1 min at 72°C	1 min at 55°C

MOP-R	F-5'CAATGCAGAAGTGCCAAGAA3' R-5'CAAGATGAAGACTGCCACCA3'	45	30 sec at 95°C	1 min at 72°C	1 min at 56°C

KOP-R	F-5'AAGGAGCACTCAATGAC3' R-5'CAGCATCTTCACCTTGACCA3'	35	1 min at 94°C	1 min at 72°C	1 min at 55°C

DOP-R	F-5'GGACGCTGGTGGACATC3' R-5'GGATCCCGTCTCCGAAACA3'	40	30 sec at 96°C	1 min at 72°C	30 sec at 58°C

Primers (F, forward and R, reverse) used for amplification of SSTRs, opioid receptors and β-actin genes and PCR conditions are indicated.

### Radioligand binding experiments

U266 cells were harvested by centrifugation (100 g, 5 min). The resulting pellet was resuspended in 50 mM Tris-HCl, pH 7.4 and disrupted with a Polytron (5 × 3 sec) at 4°C. The homogenate was ultracentrifuged at 100.000 g during 35 min at 4°C. Then, the pellet was resuspended in 50 mM Tris-HCl, pH 7.4 by sonication, protein concentration was determined by the Bradford method using bovine serum albumin (BSA) as standard and the homogenate was ultracentrifuged as before. The final pellet, which corresponds to the crude membrane fraction, was dispersed by sonication in binding buffer (50 mM HEPES, 5 mM MgCl_2_, 1 mM CaCl_2_, 0.2% (w/v) BSA, pH 7.4 for [^125 ^I-Tyr^0^] somatostatin (Phoenix Pharmaceuticals) binding or in 50 mM Tris-HCl, pH 7.4 for [^3^H]diprenorphine (NEN PerkinElmer) binding) at a final concentration of 4–6 mg/mL. Proteins (200–300 μg) were incubated with desired concentrations of the radioligand (from 0.01 to 0.5 nM of [^125 ^I-Tyr^0^] somatostatin and from 0.5 to 20 nM of [^3^H]diprenorphine) in the absence (total binding) or in the presence of cold cyclo [7-aminoheptanoyl-Phe-DTrp-Lys-Thr(Bzl)] (100 nM cyclosomatostatin) or levorphanol (50 μM) (nonspecific binding) during 30 min at 37°C in 250 μL of binding buffer. Samples were then rapidly filtered on glass-fiber discs (Whatman GF/B) and washed twice with 1 mL of ice-cold washing buffer for [^125 ^I-Tyr^0^] somatostatin (500 mM NaCl, 0.1% (w/v) BSA, pH 7.4) or 10 mM Tris-HCl, pH 7.4 for [^3^H]diprenorphine. Bound radioactivity was measured in a scintillation counter. All experiments were carried out in duplicate (SSTR binding) or in triplicate (opioid receptor binding) and repeated at least three to four times.

### Western blot analysis

Cells were harvested by centrifugation (100 g, 5 min) and the resulting pellet was suspended in lysis buffer (10 mM Tris-HCl, 1 mM EDTA, 0.1% (v/v) Triton-X100, pH 7.4) and sonicated at 4°C. Supernatants were cleared by centrifugation (20.000 g, 20 min at 4°C) and protein concentrations were determined by the Bradford assay. Equal amounts of proteins were resolved on 10% (w/v) acrylamide gels by SDS-PAGE and transferred onto a nitrocellulose membrane. After incubating for 1 h in blocking buffer (phosphate-buffered saline (PBS), 5% (w/v) nonfat dry milk or PBS, 0.1% (v/v) Tween-20 (PBS-T), 5% (w/v) nonfat dry milk), membranes were immunoblotted with a 1:1000 dilution of rabbit anti-KOP-R (Abcam) or anti-DOP-R (Oncogene) or with a 1:2000 dilution of the rabbit anti-MOP-R (Abcam) antibody overnight at 4°C. After washing in PBS or PBS-T, nitrocellulose sheets were incubated with a 1:2000 dilution of peroxidase-conjugated anti-rabbit IgG (Sigma Aldrich) for 3–4 h in the blocking buffer. Opioid receptors were revealed using the enhanced chemiluminescence system (PerkinElmer Life Sciences) with human placenta, SK-N-BE and SH-SY5Y cells as positive controls.

### Cell viability assay

Cell viability was determined using CellTiter 96^® ^AQ_ueous _One Solution Cell Proliferation Assay (Promega) according to the manufacturer's instructions. All experiments were done in culture medium containing FCS. The day before agonist treatment, cells were allowed to proliferate in fresh culture medium. After assuring that the viability was more than 90%, cells were seeded at a density of 3 × 10^4 ^cells/well in 96-well microtiter plates. U266 cells were exposed or not (control) in the presence of various concentrations of octreotide (Oct) or Sst alone or combined with their antagonist cyclosomatostatin (Css) at 10 μM for various times (24, 48 or 72 h). Cells were also treated with a combination of Sst and morphine (opioid agonist). Each condition was realised in triplicate and compared to control cells performed in sextuplet. The optical densities were measured at 492 nm and corrected by subtracting the average absorbance from wells containing cell-free medium (blank). Results are normalised compared to control cells and the percentage of viable cells is expressed according to the following formula: ((ligand treated cells - blank)/(control cells - blank)) × 100.

### Apoptosis and cell cycle analysis

U266 cells were prepared as described above except that cells were seeded into 6-well plates at a density of 6 × 10^4 ^cells/well. In order to observe a putative potentiation of apoptosis with SSTRs, U266 cells were pretreated or not (control) with 0.1 ng/mL of the agonistic Fas antibody 7C11 alone or combined with Sst or Oct for 24, 48 or 72 h.

For the cell cycle analysis, cells were pelleted by centrifugation (100 g, 5 min) and fixed in 80% (v/v) ice-cold ethanol and the pellet was incubated in phosphate-buffered saline (PBS) containing 100 μg/mL RNAse A (Qiagen) and 20 μg/mL PI (Sigma-Aldrich) for 30 min at 37°C. Cell sorting was performed using the Epics Altra (Beckman Coulter). Gated events (2 × 10^4^), except doublets and aggregates, were acquired for each sample and the results were analyzed with Wincycle^® ^software.

For apoptosis detection, cells were pretreated or not (control) as described above for cell cycle analysis. At the end of the treatment, cells were rapidly centrifuged and apoptosis was assessed using AnnexinV-FITC Apoptosis Detection Kit II "AnnexinV-PI" (BD Pharmingen) as described by the manufacturer. Samples were analysed on Epics Altra (Beckman Coulter).

### Statistical Analysis

All results are expressed as the mean ± S.E.M of n experiments. ANOVA followed by the Bonferroni-Dunn test was used to determine the statistical significance (Statview^®^).

## Results

### Expression of SSTRs and opioid receptors in malignant hemopathy cell lines

**Figure 1:**
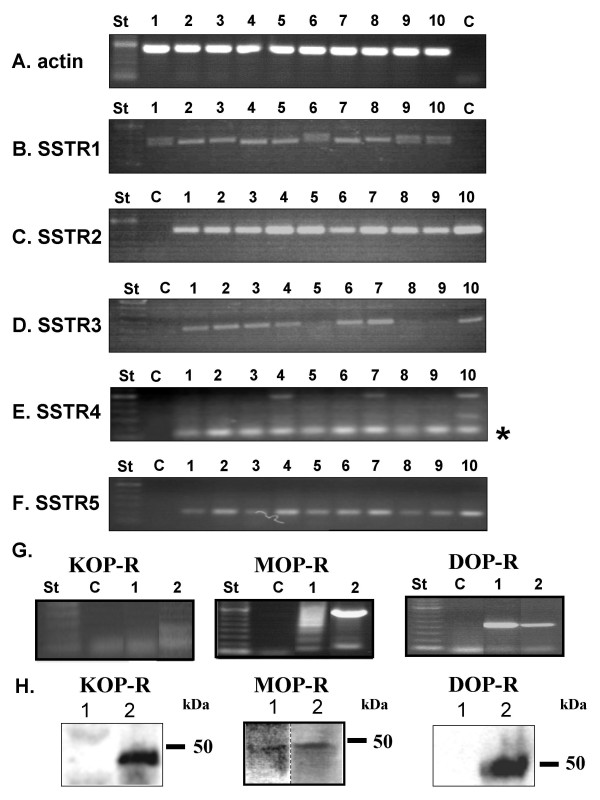
**Expression of SSTRs and opioid receptors in malignant haematological cell lines**. **A-F**, RNAs were extracted from various hemopathy cell lines, reverse transcribed, and cDNAs encoding for SSTR1 to 5 were amplified by PCR. PCR products were separated on agarose gel and stained with ethidium bromide. **St**: 100 pb ladder, **1**: Jurkat, **2**: Nalm6, **3**: RPMI-8226, **4**: Ramos, **5**: MCF-7, **6**: NCI-H929, **7**: LP-1, **8**: SH-SY5Y, **9**: 697, **10**: U266, **C**: negative control. * corresponds to the band of the expected size. **G**, opioid receptors (KOP-, DOP- and MOP-R) were amplified by PCR. **St**: 100 pb ladder, **1**: U266, **2**: SH-SY5Y, **C**: negative control. **H**, expression of opioid receptors (KOP-, DOP- and MOP-R) was studied by western-blot in U266 cells (lane 1) and in positive controls (lane 2): human placenta (KOP-R), SH-SY5Y (MOP-R) and SK-N-BE cells (DOP-R). Data are representative of three independent experiments.

To investigate SSTRs and opioid receptors expression in various malignant haematological cell lines, total RNA extraction was performed from the pre-B leukaemia cell lines 697 and Nalm6, the MM cell lines RPMI-8226, U266, LP-1, NCI-H929, the Burkitt lymphoma cell line Ramos and the T lymphoma cell line Jurkat, followed by RT-PCR. The human neuroblastoma cell line SH-SY5Y and the breast carcinoma cell line MCF-7 were used as positive controls for SSTRs expression [21,22]. The ubiquitously expressed β-actin gene was used as control (Figure 1A). The PCR for SSTR1 was positive in all cell lines but doublet PCR products could be detected in Jurkat, NCI-H929, 697 and U266 cell lines (Figure 1B) as previously described in hepatocellular carcinoma [23]. When examining SSTR2 mRNAs expression, we found the presence of a single band in all cell lines and in SH-SY5Y and MCF-7 cells (Figure 1C). SSTR3 mRNAs were detected in Jurkat, Nalm6, RPMI-8226, Ramos, NCI-H929, LP-1 and U266 (Figure 1D) while the two other SSTR subtypes were amplified in all cell lines that we examined (Figure 1E and 1F). As a preliminary work performed on U266 cells suggested that they contain opioid receptors, we further characterised their subtypes. In the U266 cells, we were able to detect mRNAs encoding for the DOP- and MOP-R but not KOP-R while all the three opioid receptors were observed in the SH-SY5Y cells (Figure 1G). It's worthy to note that several bands were amplified in U266 cell line for MOP-R, probably reflecting the presence of alternative splicing of this gene as previously reported [24]. In western-blot experiments, MOP-, DOP- and KOP-R were detected in positive controls (SH-SY5Y, SK-N-BE and human placenta, respectively) [25-28] whereas only the MOP-R was evidenced in U266 cells (Figure 1H). Furthermore, SSTRs and opioid receptors were also revealed by using their ability to bind their prototypic ligands, [^125 ^I-Tyr^0^] somatostatin and [^3^H]diprenorphine, respectively (Table 2).

**Table 2:** U266 cells express opioid and somatostatin binding sites.

**[Diprenorphine] (nM)**	**CPM**	**[Somatostatin] (nM)**	**CPM**
0,5	44 ± 32	0,025	139 ± 66

1	127 ± 84	0,05	506 ± 313

2,5	157 ± 90	0,076	628 ± 92

5	197 ± 78	0,1	677 ± 326

10	552 ± 276	0,25	987 ± 483

20	2746 ± 1382	0,5	2464 ± 869

Crude membrane fraction was incubated with [^125^I-Tyr^0^] somatostatin or [^3^H]diprenorphine as described in materials and methods. Data represent mean ± S.E.M. (n = 3–4) of specific binding expressed in CPM.

Thus, the U266 cell line represents a suitable model for exploring putative interactions between somatostatin and opioid receptors to modulate cellular proliferation and apoptosis [29-33].

### Effect of SSTR and opioid agonists on U266 cell viability

**Figure 2:**
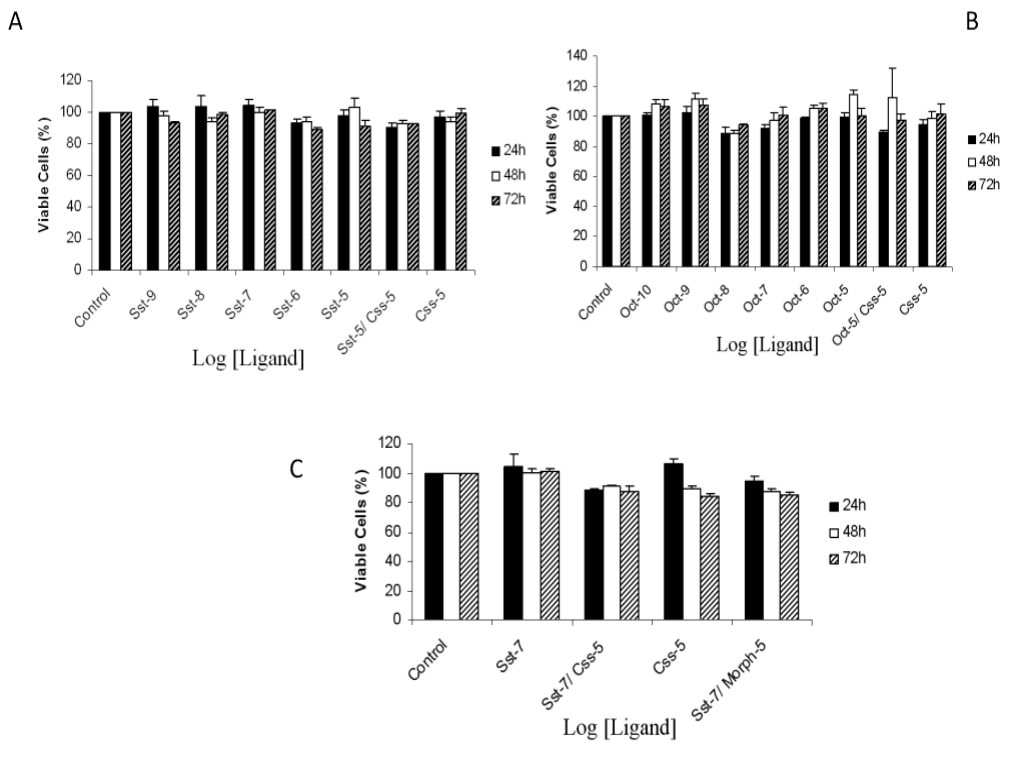
**Effect of Sst, Oct and Morph on U266 cell line viability**. Exponentially growing cells were seeded and incubated for 24, 48 or 72 h with (A) somatostatin (Sst), (B) octreotide (Oct), (C) Sst alone or combined with 10 μM morphine (Morph). The SSTR antagonist cyclosomatostatin (Css) was also included. U266 cell viability was determined using the XTT assay and data were normalized to absorbance values obtained in control cells. Data are mean ± S.E.M of 5 to 7 different experiments performed in triplicate.

Cell viability was then evaluated using XTT assays. All experiments were done in culture medium containing FCS. U266 cells were treated or not (control) in the presence of either Sst or Oct, a SSTR2, 3 and 5 selective agonist [6,34], ranging from 100 pM to 10 μM during 24, 48 or 72 h. As depicted on the Figure 2A, Sst, even at high concentrations, was devoid of any significant effect on cell viability at 24, 48 or 72 h pretreatment. When cells were exposed to a selective SSTR antagonist, cyclosomatostatin (Css), alone or in combination with Sst, no significant effect was detected. Stimulation of SSTR2, 3 and 5 by Oct (100 pM to 10 μM) alone or in combination with 10 μM of Css for 24, 48 or 72 h was unable to promote any significant modification of cell viability (Figure 2B).

Next, we evaluated the potential interactions between opioid and somatostatin receptors. U266 cells were exposed or not (control) either to Sst alone, to a combination of Sst plus 10 μM morphine (Morph) or Css, but still no modification of U266 cell viability was noted after 24, 48 or 72 h (Figure 2C).

### Effects of Sst and Oct on cell cycle distribution in U266 cells

**Figure 3:**
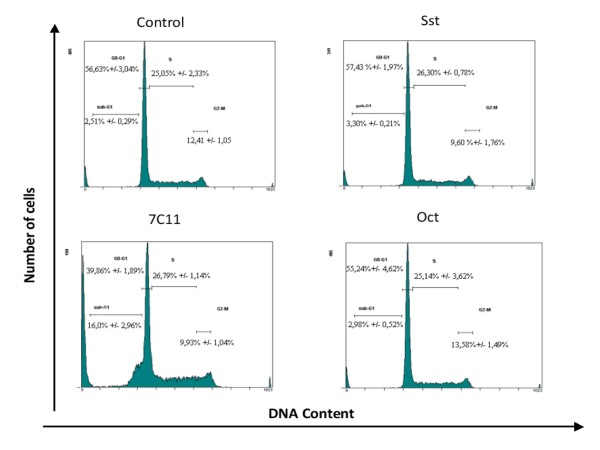
**Cell cycle distribution of U266 cells after SSTR stimulation**. Exponentially growing cells were incubated with 10 μM Sst or Oct, or with 0.1 mg/mL 7C11 (agonistic Fas antibody) for 72 h. DNA content analysis was done after PI staining of ethanol-permeabilized cells. % of each cell cycle phase are summarized in the Table 2. Data shown are representative of 3 independent experiments.

We confirmed by using an alternative method, that SSTR agonists were ineffective to regulate U266 cell proliferation. Distribution in the cell cycle of control or agonist-pretreated U266 cells was determined after PI staining by flow cytometry. A low (10 nM) or a high concentration (10 μM) of Sst or Oct alone, or in combination with Css were selected and cells were exposed during 24, 48 or 72 h. A representative experiment is depicted in the Figure 3 showing that neither Sst (10 μM) nor Oct (10 μM) were able to promote changes in cell cycle distribution compared to control cells after 72 h. Similar data were obtained for 24 and 48 h pretreatment (data not shown). The percentage of each phase was determined for control or agonist-pretreated cells and these data are summarised in the Table 3.

**Table 3:** Cell cycle distribution of U266 MM cell line treated with SSTR ligands and 7C11

**Treatment**	**G0-G1 (%)**	**S (%)**	**G2-M (%)**	**Sub-G1 (%)**
Control	56,6 ± 3,0	25,1 ± 2,3	12,4 ± 1,1	2,5 ± 0,3

Sst 10 μM	57,4 ± 2,0	26,3 ± 0,8	9,6 ± 1,8	3,3 ± 0,2

Css 10 μM	60,8 ± 2,4	20,7 ± 2,4	11,2 ± 0,1	3,7 ± 0,8

Sst 10 μM/Css 10 μM	57,3 ± 2,2	26,2 ± 0,9	10,0 ± 2,5	2,9 ± 0,4

7C11	39,9 ± 1,5*	26,8 ± 1,1	9,9 ± 1,0	16,0 ± 0,9*

7C11/Sst 10 μM	40,3 ± 1,8*	27,2 ± 0,4	8,6 ± 1,1	14,0 ± 0,7*

7C11/Sst 10 μM/Css 10 μM	38,3 ± 3,3*	27,3 ± 1,0	8,9 ± 0,8	12,0 ± 1,1*

Oct 10 μM	55,2 ± 4,6	25,1 ± 3,5	13,6 ± 1,5	3,0 ± 0,5

Oct 10 μM/Css 10 μM	55,6 ± 4,7	24,9 ± 3,6	12,6 ± 1,6	4,0 ± 0,8

7C11/Oct 10 μM	43,1 ± 0,5*	27,2 ± 1,7	12,2 ± 1,5	13,6 ± 1,9*

7C11/Oct 10 μM/Css 10 μM	41,9 ± 0,8*	26,4 ± 2,6	8,1 ± 0,4	18,2 ± 4,6*

U266 cells were pretreated or not (control) with Sst, Oct, Css or the agonistic Fas antibody 7C11 (7C11) for 72 h. Cells were stained with PI, analyzed by flow cytometry and each fraction of the cell cycle was determined using Wincycle^®^. Data are mean ± S.E.M. of 3 independent experiments. *, ANOVA followed by Bonferroni-Dunn (p < 0.05), statistically significant differences compared to control cells.

### Effect of somatostatin and opioid receptors activation on apoptosis

**Figure 4:**
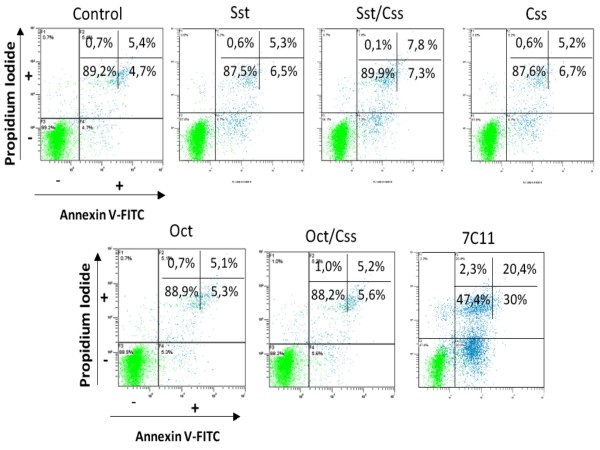
**Apoptosis study of U266 cells after SSTR and Fas receptor activation**. Exponentially growing cells were incubated with 10 μM Sst, Oct, Css alone or combinated, or with 0.1 mg/mL 7C11 (agonistic Fas antibody) for 72 h. Cells were stained with annexinV-FITC and PI and analyzed by fluorescence-activated cell sorting to quantify apoptosis. Data shown are representative of 6 independent experiments.

Previous studies demonstrated that Sst analogs induced apoptosis in several cell models [35-38]. As MM cells are characterised by apoptosis resistance (see for review [39]), we aimed to explore whether Sst or Oct would sensitise the extrinsic cell death pathway. U266 cells were incubated for 24, 48 or 72 h with 0.1 mg/mL of the agonistic Fas antibody 7C11 alone or in combination with SSTR ligands. In 7C11-treated cells and after 72 h pretreatment, we observed a significant increase in sub-G1 cell population indicating the occurrence of apoptosis that was associated with a reduction of the G0-G1 fraction (Figure 4 and Table 3). Combination of the 7C11 antibody with Sst, Oct, or Css did not produce additional change compared to 7C11-treated cells (Table 3). Identical results were obtained upon 24 and 48 h exposure but with a less marked effect of 7C11 (data not shown).

U266 apoptosis was quantified using annexin V-FITC and PI staining by flow cytometry. When cells were treated for 72 h in the presence of Sst, Oct or Css alone or in combination, no significant modification of the percentage of viable (annexin V^-^/PI^-^), necrotic (annexin V^-^/PI^+^), early apoptotic (annexin V^+^/PI^-^) or late apoptotic cells (annexin V^+^/PI^+^) could be detected compared to control U266 cells (Figure 4). In contrast, 7C11 was able to promote apoptosis as shown by an increase of both annexin V^+^/PI^- ^and annexin V^+^/PI^+ ^cells with a concomitant reduction of viable cells (Figure 4). When we assessed the combination of 7C11 with Sst or Oct, alone or associated with Css, no further modulation of apoptosis could be observed (data not shown).

## Discussion

SSTRs are widely expressed within the central nervous system, the endocrine system, the gastro-intestinal tract (see for review [40]) but also in immune cells (see for review [9]). Normal B and T cells were reported to exclusively express SSTR3 [13]. In the current study, we observed that all human MM cell lines express the five SSTR subtypes. Our data are in agreement with those obtained by Georgii-Hemming and collaborators [41] who observed only the expression of SSTR2, 3 and 5 by using binding and RT-PCR experiments. We also confirmed in binding studies using [^125 ^I-Tyr^0^] somatostatin that U266 cells express a substantial amount of SSTRs. The different patterns of SSTRs expression between malignant and non-tumoral B cells suggest that these GPCRs would play a role in oncogenesis or would be a specific marker of malignant hemopathies. This hallmark is not restricted to B cells as we also noticed that the human T cell leukaemia Jurkat expresses the five different SSTR subtypes while SSTR3 is mainly found in normal T lymphocytes [13]. We also found that pre-B cells (Nalm-6 and 697), a B-cell lymphoma (Ramos) and MM cell lines (LP-1, RPMI, U266 and NCI-H929) express also the five different SSTR subtypes. This suggests that a) the SSTRs expression is found along the B cell differentiation stages b) SSTRs expression is not modulated during this process b) SSTRs expression pattern is not a marker for B cell differentiation.

Sst and its analogs have been demonstrated to negatively regulate tumor cell proliferation (see for review [42]) and have been used in inoperable patients where neuroendocrine tumours stabilization or shrinkage can be obtained [43]. However, in other cancers such as hepatocellular carcinoma, the clinical benefit of Oct is not evidenced even in positive Oct scintigraphy patients [44]. To our knowledge, only one study examined the effects of Sst and Oct in MM cell lines and showed a strong decrease of viable cells after 48 h Oct exposure [41]. This is in marked contrast with our data since either Sst or Oct were unable to affect cell proliferation of the U266 cell line. Such discrepancies should be explained by the use of different clones of the U266. We can also hypothesize that our U266 cells would express SSTRs with opposite effects on proliferation. SSTR2 and 5 were reported to inhibit cell proliferation by phosphotyrosine phosphatase (PTP) activation and inhibition of calcium channels, respectively [42,45]. In contrast, SSTR4 were shown to activate the MAPK cascade and promoting proliferation [46]. So, no effect on proliferation would be observed upon co-activation of those SSTRs. Discrepancies between our study and the one of Georgii-Hemming and collaborators [41] about cellular viability should also be due to the presence or the absence of serum in the culture medium. However, we can rule out such explanation since we observed no effect upon SSTR agonists when experiments were conducted in serum-free culture medium (data not shown).

Anti-tumoral activity of Sst or its analogs are also due to pro-apoptotic effects (see for review [47]). In two MM cell lines U266 (current study) and LP-1 (data not shown), we observed that neither Sst nor Oct promote apoptosis in our experimental conditions. This was illustrated by the lack of sub-G1 peak in cell cycle assay and the absence of labelling in annexin V/PI experiments. In contrast, Georgii-Hemming et al. showed that in three MM cells (HL-407L, HL-407E and U-1958) Oct induced a weak increase in annexin V/PI staining suggesting that SSTRs could promote apoptosis [41] but the U266 cell line was not investigated. Sharma et al. first described the role of SSTR3 in apoptosis when expressed in Chinese hamster ovary cells and demonstrated that Oct promotes dephosphorylation of wild-type p53 which leads to DNA fragmentation [35]. Even in the absence of apoptosis, we can not rule out that SSTRs are not coupled to apoptotic pathways since U266 was shown to express the anti-apoptotic protein Bcl-2 [48].

MM cells were reported to express death receptors, including Fas (CD95), which triggers extrinsic apoptotic pathway [49]. When activated by the agonistic Fas antibody 7C11, this receptor produces apoptosis. After activation of SSTRs either by the endogenous agonist or Oct, we were unable to detect any enhancement of Fas-induced apoptosis. This is in contrast with previous data obtained in the pancreatic cancer BxPC-3 cells [50]. Indeed, SSTR2 was shown to up-regulate TNF-related apoptosis-inducing ligand (TRAIL) receptors, DR4 and TNFRI that trigger death first, by activating caspase 8 and second by down-regulating the anti-apoptotic mitochondrial protein Bcl2.

Opioid receptors are also expressed in immune cells [51] in which they promote apoptosis by regulating Fas expression [31]. These GPCRs were shown to heterodimerize with SSTRs [52] and we hypothesized that co-treatment with opioids and Sst or Oct would activate signalling pathways leading to apoptosis. In the current study, we demonstrated by molecular experiments and western blot that U266 cells express MOP-R that are able to bind a prototypical ligand [^3^H]diprenorphine. When morphine (a MOP-R "selective" agonist) was used alone, no evidence for apoptosis was detected. Similar results were obtained when both opioid and somatostatin receptors were co-activated. While morphine and ethylketocyclazocine were reported to interact with SSTRs in the opposum kidney cells and HepG2 cell line, respectively, and promote cell growth inhibition [53,54], our data rule out such conclusions in our cellular model.

## Conclusion

In conclusion, we demonstrated that the human MM cell line U266 expresses both SSTRs and the MOP-R. However, their stimulation by Sst, Oct or morphine alone or in combination fails to induce cell cycle modifications and apoptosis in U266 cells. While we demonstrated that Oct has no effect on the myeloma cell lines U266 and LP-1 (data not shown), we can not exclude that such targeted treatment would be ineffective in patients.

## Competing interests

The authors declare that they have no competing interests.

## Authors' contributions

CK: acquisition, analysis and interpretation of data.

TC: carried out the molecular study

BS: involved in drafting the manuscript

PJ: involved in drafting the manuscript

SA: conception of project, analysis and interpretation of data

## Authors' information

CK: Ph.D. student. In addition CK is a recipient of the Ministère de l'enseignement supérieur et de la recherche

TC: M.D. student

BS: Ph.D.

PJ: M.D., Ph. D.

SA: Ph.D.
